# P-1026. Antimicrobial Prophylaxis in Temporary Mechanical Circulatory Support

**DOI:** 10.1093/ofid/ofaf695.1222

**Published:** 2026-01-11

**Authors:** Alainee Miller, Ty Drake, Melanie Madorsky, Phillip Weeks

**Affiliations:** Memorial Hermann-Texas Medical Center, Houston, Texas; Memorial Hermann - Texas Medical Center, Houston, Texas; Memorial Hermann-Texas Medical Center, Houston, Texas; Memorial Hermann-Texas Medical Center, Houston, Texas

## Abstract

**Background:**

Mechanical circulatory support (MCS) devices are associated with a substantial risk of infection due to the presence of surgical wounds, intra-vascular lines, and external drains. These risks often prompt healthcare professionals to utilize prolonged prophylactic antibiotics during device support. Current evidence exploring the risks and benefits of prophylactic antibiotic use in this population is limited.

**Methods:**

A single-center retrospective cohort study was conducted to evaluate the impact of prolonged antibiotic prophylaxis in patients receiving temporary MCS devices. Included devices were intra-aortic balloon pumps (IABPs) and micro-axial percutaneous ventricular assist devices (e.g., Impella) with an insertion duration of more than 48 hours. Patients with extracorporeal membrane oxygenation (ECMO) or neutropenia (absolute neutrophil count less than 1500 cells/μL) were excluded. Patients were categorized into short duration (antibiotics for 25% or less of device time) and extended duration (antibiotics for 75% or more of device time) prophylaxis groups, matched 1:1 by device duration (48 hours to 7 days, 8 to 20 days, and 21 days or more). Outcomes included catheter-related bloodstream infections, positive cultures from any site, *Clostridioides difficile* infection, and all-cause mortality.

**Results:**

A total of 278 MCS devices were analyzed. IABPs accounted for 79.9% of devices. No significant differences were observed in bloodstream infection rates (2.2% vs. 1.4%; p=0.7), *C. difficile* infections (1.4% vs. 0%; p=0.2), and positive culture rates (7.2% vs. 7.2%; p=1). However, the extended duration group was associated with significantly lower survival (hazard ratio: 1.85; 95% CI: 1.1-3.2). Device duration was not associated with infection risk or mortality.

**Conclusion:**

Extended prophylactic antibiotic use during temporary MCS device support was not associated with reduced infection rates or improved survival. On the contrary, it was associated with a lower survival rate in patients with MCS devices. Prospective studies are needed to define optimal antimicrobial strategies in this high-risk population.

**Disclosures:**

All Authors: No reported disclosures

